# Plastid-Localized EMB2726 Is Involved in Chloroplast Biogenesis and Early Embryo Development in *Arabidopsis*

**DOI:** 10.3389/fpls.2021.675838

**Published:** 2021-07-23

**Authors:** Chuanling Li, Jian-Xiu Shang, Chenlei Qiu, Baowen Zhang, Jinxue Wang, Shuo Wang, Yu Sun

**Affiliations:** Hebei Key Laboratory of Molecular and Cellular Biology, Ministry of Education Key Laboratory of Molecular and Cellular Biology, Hebei Collaboration Innovation Center for Cell Signaling, College of Life Sciences, Hebei Normal University, Shijiazhuang, China

**Keywords:** EF-Ts, embryo development, chloroplast, *EMB2726*, *Arabidopsis*

## Abstract

Embryogenesis is a critical developmental process that establishes the body organization of higher plants. During this process, the biogenesis of chloroplasts from proplastids is essential. A failure in chloroplast development during embryogenesis can cause morphologically abnormal embryos or embryonic lethality. In this study, we isolated a T-DNA insertion mutant of the *Arabidopsis* gene *EMBRYO DEFECTIVE 2726* (*EMB2726*). Heterozygous *emb2726* seedlings produced about 25% albino seeds with embryos that displayed defects at the 32-cell stage and that arrested development at the late globular stage. EMB2726 protein was localized in chloroplasts and was expressed at all stages of development, such as embryogenesis. Moreover, the two translation elongation factor Ts domains within the protein were critical for its function. Transmission electron microscopy revealed that the cells in *emb2726* embryos contained undifferentiated proplastids and that the expression of plastid genome-encoded photosynthesis-related genes was dramatically reduced. Expression studies of *DR5:GFP*, *pDRN*:*DRN-GFP*, and *pPIN1:PIN1-GFP* reporter lines indicated normal auxin biosynthesis but altered polar auxin transport. The expression of *pSHR:SHR-GFP* and *pSCR:SCR-GFP* confirmed that procambium and ground tissue precursors were lacking in *emb2726* embryos. The results suggest that EMB2726 plays a critical role during *Arabidopsis* embryogenesis by affecting chloroplast development, possibly by affecting the translation process in plastids.

## Introduction

Higher plant development is a complex process that begins with embryogenesis. In *Arabidopsis*, embryogenesis starts from the asymmetric division of a zygote to generate a large basal cell and a small apical cell (Bayer et al., 2017). The basal cell divides horizontally, and the resulting uppermost cell, the hypophysis, and develops into the root meristem. Meanwhile, the apical cell undergoes two vertical symmetric divisions and one horizontal symmetric division, after which an asymmetric periclinal division separates the protoderm, precursor of the epidermis, and from the inner cells. The protodermal cells then divide anticlinally, while the inner cells divide longitudinally, and the embryo enters the 32-cell, early globular, and stage. At this point, precursor cells of all tissue types are formed, but the actual initiation of different tissues occurs at the late globular stage. The cells in the upper tier initiate formation of the cotyledon and shoot apical meristem, whereas the cells in the lower tier establish the ground tissue and procambium. Eventually, a well-defined tissue pattern is established at the heart stage (Lau et al., 2012; Wendrich and Weijers, 2013; Ten Hove et al., 2015). The globular to heart stages are the most critical stages during embryogenesis; more than 50% of mutants with strong embryo phenotypes show defects at these stages (Meinke, 2020).

In *Arabidopsis*, two important events occur at the globular stage: chloroplast differentiation from proplastids and chlorophyll biosynthesis (Tejos et al., 2010). It has been proposed that photosynthesis is important for avoiding hypoxic stress and promoting energy production in developing embryos and seeds (Borisjuk and Rolletschek, 2009). Photosynthesis also produces energy-rich compounds, which can be used for the biosynthesis of storage products for the embryo (Puthur et al., 2013). However, most photosynthesis-related mutants are capable of generating weak seedlings with albino cotyledon/leaves (Motohashi et al., 2001; Huang et al., 2009), and Liu et al. (2017) showed that *Arabidopsis* embryos are still able to develop into mature seeds under light deprivation conditions, demonstrating that the photosynthetic ability of chloroplasts is not required for embryogenesis. In addition to their photosynthetic functions, chloroplasts, and non-photosynthetic plastids are important sites for the synthesis of various biomolecules, such as amino acids, fatty acids, and precursors of several plant hormones (Hsu et al., 2010; Allorent et al., 2015), which are critical for maintaining plant growth and development. For example, tryptophan, the precursor of auxin, is synthesized in plastids (Radwanski and Last, 1995). Also, the first step of fatty acid biosynthesis occurs in plastids and is catalyzed by heteromeric acetyl-CoA carboxylase, which contains a subunit (accD) that is encoded by the plastid genome (Sasaki and Nagano, 2004). Moreover, plastids can regulate nuclear gene expression to coordinate cellular activities through retrograde signaling (Inaba and Ito-Inaba, 2010; Hernández-Verdeja and Strand, 2018).

In a screen for nucleus-encoded plastid-localized proteins, Bryant et al. (2011) identified 119 genes that were required for embryogenesis. Recently, Meinke (2020) presented more than 500 *Arabidopsis* genes that function during embryogenesis; notably, 30–40% of them are related to plastid development. Detailed analyses have also provided direct evidence showing that defects in chloroplast biogenesis lead to defects in embryo development (Lu et al., 2014; Chen et al., 2015, 2018; Ye et al., 2017). As semi-autonomous organelles, plastids possess their own translational process that has a significant impact on plastid biogenesis and embryo development. For example, mutations in the plastid ribosomal protein L21 (Yin et al., 2012), plastid-localized translation elongation factor G (Ruppel and Hangarter, 2007), and ribosome recycling factor (Wang et al., 2010) cause undeveloped plastids and embryonic lethality. EMB2726, a homolog of the plastid-localized polyprotein of elongation factor (EF)-Ts (PETs) protein from *Chlamydomonas reinhardtii* (Belign et al., 2004), contains two EF-Ts domains at its carboxyl end. It had been noticed that its homozygous mutant embryos were arrested at the globular stage of development (Tzafrir et al., 2004). During translation in prokaryotes, EF-Ts, a guanine nucleotide exchange factor, is required to facilitate the rapid conversion of EF-Tu⋅GDP to EF-Tu⋅GTP and to maintain the rate of protein synthesis. As a prokaryote-derived endosymbiotic organelle, the plastid possesses a translation system that is very similar to that of bacteria, implying that EMB2726 is involved in translation in plastids. However, experimental evidence for the function of EMB2726, especially during embryogenesis, is highly lacking.

In this study, we identified a T-DNA knockout mutation of *EMB2726.* About 25% of the seeds in *emb2726/*+ siliques were albino with embryos that exhibited an initial phenotype at the 32-cell stage and arrested development at the globular stage, suggesting that *EMB2726* plays an important role in embryo development. EMB2726 was localized exclusively in plastids from the start of embryogenesis, and the embryos in albino seeds from *emb2726/*+ siliques contained only undifferentiated proplastids. Deletion of the EF-Ts domains from EMB2726 resulted in plants with phenotypes similar to those of null allele mutants, indicating that EMB2726 is involved in the translation in plastids. Collectively, the results provided evidence supporting that EMB2726 functions as an EF-Ts during translation in plastids to regulate chloroplast biogenesis and embryo development.

## Materials and Methods

### Plant Growth Conditions and Constructs

*Arabidopsis thaliana* ecotype Columbia-0 (Col-0) was used as the wild-type (WT) control in this study. Seedlings were grown in a growth room under 16 h of light/8 h of darkness (90 μmol m^–2^ s^–1^ intensity) at 22°C and 55% relative humidity.

To generate the *emb2726-5/*+ and *emb2726-6/*+ mutations, an egg cell-specific promoter-controlled CRISPR/Cas9 system was used according to Wang et al. (2015).

To produce the native promoter-driven constructs *pEMB2726:EMB2726-GUS* and *pEMB2726:EMB2726-YFP*, the *EMB2726* genomic sequence (with 2,032 base pairs of sequence upstream of the ATG as a promoter) was amplified by PCR using the primers listed in Supplementary Table 1 and cloned into the donor vector pCR^TM^8/GW/TOPO^TM^ (Invitrogen, Waltham, MA, United States) following the instructions of the manufacturer. Then, the cloned sequence was subcloned into the binary vectors, pMDC163 (Curtis and Grossniklaus, 2003) and pEarleyGate-TW1 (Wang et al., 2016), using Gateway^TM^ LR Clonase^TM^ II Enzyme Mix (Invitrogen, Waltham, MA, United States).

The abovementioned expression constructs were introduced into the *Agrobacterium tumefaciens* strain GV3101 and transformed into Col-0or *emb2726-4/*+ plants using the floral dip method.

### Microscopy and Image Analysis

For embryo observation, siliques at 2–6 days after pollination (DAP) were sliced open and immersed in 4% glutaraldehyde in phosphate-buffered saline (PBS; 8 g/L NaCl,0.2 g/L KCl, 1.44 g/L Na_2_HPO_4_, and 0.24 g/L KH_2_PO_4_, pH 7.5). They were then vacuum-infiltrated for 15 min and kept at room temperature overnight. The seeds were then removed from the fixed siliques under a stereomicroscope and cleared using a chloral hydrate:glycerol:water solution (8:1:2, w/v/v) based on the study of Berleth and Jürgens (1993). The cleared seeds were observed by differential interference contrast microscopy using a Zeiss Axio Imager A microscope equipped with a AxioCam MRc5 camera (Zeiss, Jena, Germany).

For fluorescence microscopy, leaf epidermal samples or embryos were observed under a Zeiss LSM 710 confocal laser microscopy system with filters for yellow fluorescent protein (YFP; excitation, 514 nm; emission 519–568 nm), green fluorescent protein (GFP; excitation, 488 nm; emission 499–600 nm), and chlorophyll autofluorescence (excitation, 488 nm; emission, 650–675 nm).

### β-Glucuronidase (GUS) Histochemical Staining

Tissues from homozygous *pEMB2726:EMB2726-GUS* transgenic seedlings were vacuum-infiltrated for 30 min in a GUS staining solution (1 mg/ml of 5-bromo-4-chloro-3-indolyl-β-d-glucuronide, 50 mM NaH_2_PO_4_, 50 mM Na_2_HPO_4_, 10 mM EDTA-Na_2_, 0.5 mM K_3_Fe[CN]_6_,0.5 mM K_4_Fe[CN]_6_, and 0.1% Triton X-100) and stained for 1 h (7-day-old seedlings and rosette leaves) or 5 h (flowers, inflorescences, siliques, and seeds) at 37°C. The stained tissues were cleared with 70% ethanol and photographed.

### RNA Extraction and Quantitative Reverse Transcription (qRT)-PCR

Total RNA was isolated from green or white seeds in *emb2726-4/*+ siliques at 7 DAP using an Eastep^®^ Super Total RNA Extraction Kit (Promega, Madison, WI, United States) following the instructions of the manufacturer. Complementary DNA (cDNA) was synthesized from 0.5 to 1 μg of total RNA using the Revert Aid First Strand cDNA Synthesis Kit (Thermo Fisher Scientific, Waltham, MA, United States) with oligo (dT)_16_ primer. Quantitative PCR was performed using SYBR Premix Ex Taq (Takara Bio Inc., Otsu, Japan). The sequences of the gene-specific primers used are listed in Supplementary Table 1. *PP2AA3* was used as an internal control.

### Transmission Electron Microscopy

Green and white seeds from *emb2726-4/*+ siliques at 5 DAP were fixed, embedded, and sectioned according to the method described by Hyman and Jarvis (2011) with some modifications. In brief, the seeds were fixed in 4% glutaraldehyde/PBS (pH 6.9) for 2 h by vacuum infiltration followed by incubation at 4°C overnight. The seeds were then rinsed three times with PBS (pH 6.9) and fixed in 1% osmic acid/PBS (pH 6.9) for another 4–8 h and rinsed three more times with PBS (pH 6.9). After fixation, the seeds were dehydrated in a series of graded ethanol solutions (10, 30, 50, 70, 90, and 95%; 30 min each). The final dehydration step was done two times using 100% ethanol for 1 h each. Next, the alcohol was replaced with acetone by incubating the seeds for one time in a 1:1 acetone:alcohol solution and then for two times in 100% acetone for 30 min each. The seeds were then infiltrated using a graded series of acetone:embedding resin solutions [(v/v) 3:1, 1:1, and 1:3; 6 h each] and with 100% resin two times for 6 h each. The seeds were then transferred to capsules containing fresh embedding resin and incubated at 65°C for at least 48 h.

Ultra-thin sections were prepared using Leica Ultracut R Ultramicrotome (Leica Microsystems, Wetzlar, Germany) and collected on formvar-coated nickel grids. The sections were post-stained with saturated uranyl acetate for 15 min followed by lead citrate for 15 min before being examined and imaged with a Hitachi H-7650 (Hitachi, Ibaraki, Japan) transmission electron microscope.

## Results

### EMB2726 Is Required for Embryo Development

**FIGURE 1:**
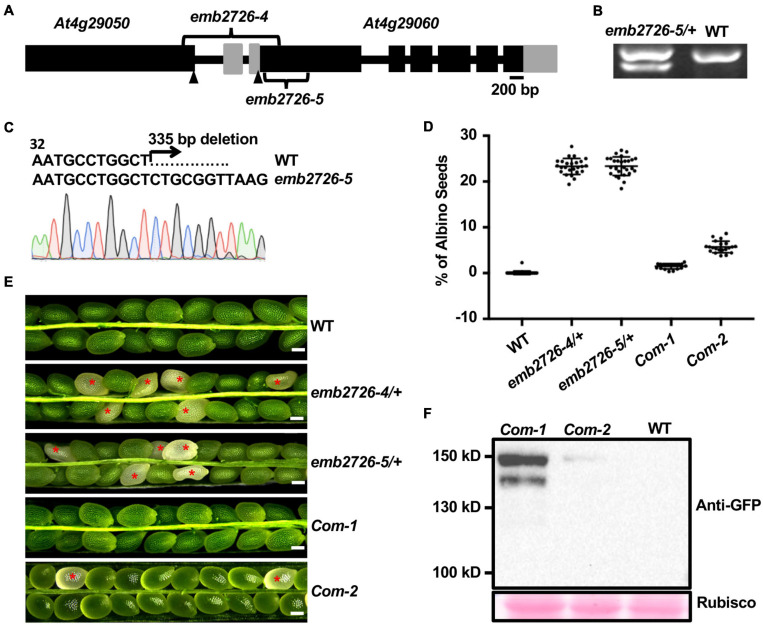
Characterization of the *emb2726* mutants and complementation lines. **(A)** Schematic diagram of the *EMB2726* gene with the deletion sites of *emb2726-4* and *emb2726-5*. Black boxes, gray boxes, and black lines represent exons, untranslated regions, and introns/intergenic sequences, respectively. Arrowheads point to the ATG (start codons) in the two neighboring genes. **(B)** Genotyping results for an *emb2726-5/*+ plant; the lower band in *emb2726-5/*+ shows the deletion allele. **(C)** Sequencing chromatogram of the mutated *emb2726-5/*+ sequence generated by CRISPR/Cas9. **(D)** Percentage of albino seeds as shown in **(E)**. **(E)** Seed phenotypes within siliques from wild-type (WT) plants, *emb2726-4/*+, *emb2726-5/*+, and two complementation lines (*Com-1* and *Com-2*). The complementation lines were generated using *p EMB2726:EMB2726-YFP* transformed into the *emb2726-4*/+ background. Scale bar = 0.2 mm. Red asterisks indicate albino seeds. **(F)** Immunoblot analysis of EMB2726-YFP expression in the two complementation lines and WT. EMB2726-YFP was detected using anti-GFP antibodies; Rubisco staining by Ponceau S was used to indicate equal loading.

In a screen for sterile mutants, we obtained a T-DNA insertion mutant that produced no homozygous progeny; the segregation ratio of WT to heterozygous offspring was 1:2 (*n* > 4,000). Subsequent analysis showed that this phenotype came from an additional insertion within the mutant that was tightly linked with the original insertion. The Thermal Asymmetric InterLaced PCR technique was applied to identify the second insertion site in the segregated line. The T-DNA left border was recovered at 348 base pairs downstream of the ATG in *At4g29060*; the right border was positioned 1,054 base pairs upstream of the start codon for *At4g29060*, within the first exon of *At4g29050*. Thus, this T-DNA insertion caused a 1.4-kb deletion between the two genes (Figure 1A). Since *At4g29060* encodes EMB2726, we suspected that the deletion at *At4g29060* was responsible for the lack of homozygous offspring from the mutant. To confirm the hypothesis, we generated another mutant for *At4g29060* using the CRISPR/Cas9 technique. This mutation contained a 335-base pair deletion from nucleotides 42 to 376 within exon 1 (Figures 1A–C), which caused a frame shift and early termination after encoding 16 amino acids. Phenotypic analyses of siliques from heterozygous plants of the T-DNA insertion mutant (named *emb2726-4*) and the CRISPR/Cas9 mutant (named *emb2726-5*) at 10 DAP showed that both mutants contained about 25% albino seeds (*n* > 1,500 for each mutant), compared to 0.07% in WT (*n* > 1,500, Figures 1D,E). Consistently, about 25% of the seeds (*n* > 1,500) from siliques at 14 DAP were highly wrinkled and dark brown in color, indicating that the embryos in the albino seeds had degenerated. Reciprocal crosses of *emb2726-4/*+ with WT plants revealed no significant defects in the transmission efficiencies of the female and male gametophytes, confirming that the lack of homozygous offspring from this mutant is due to a defect in embryogenesis (Supplementary Table 2).

For confirmation, the promoter region together with the genomic sequence of *EMB2726* was fused to *YFP* (*pEMB2726:EMB2726-YFP*) and transformed into the *emb2726-4/*+ background. T1 *emb2726-4/*+ transgenic plants with high-level protein expression produced siliques with almost no albino seeds, and the number of aborted seeds in the siliques was reduced to nearly the level in WT (Figures 1D–F). Together, these data demonstrate that loss of EMB2726 function causes an embryonic lethal phenotype.

### Homozygous *emb2726* Embryos Are Arrested at the Globular Stage of Development

**FIGURE 2:**
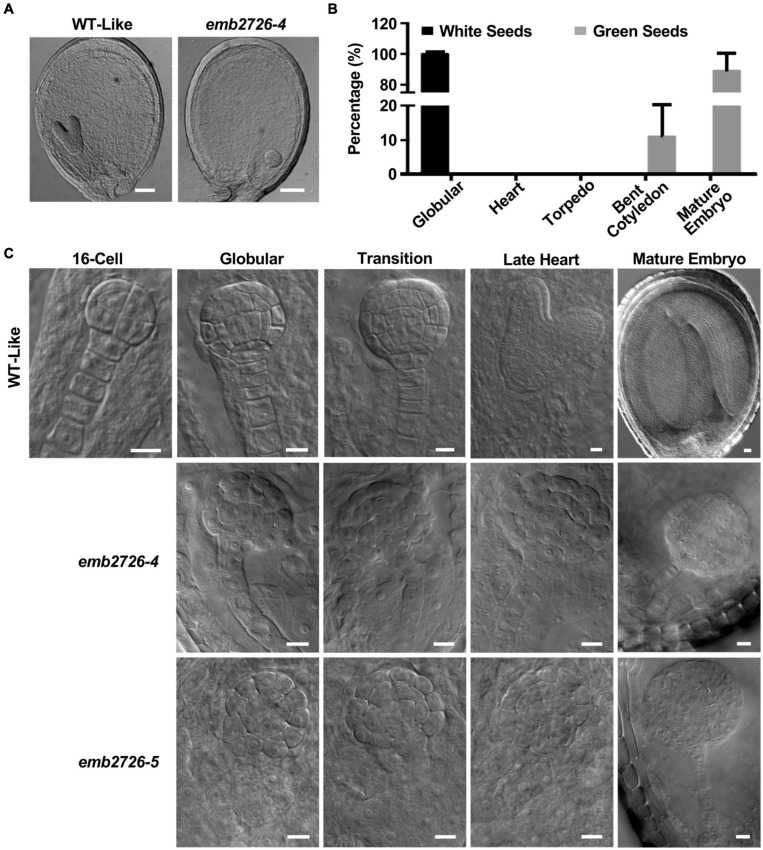
Phenotype analysis of the *emb2726* mutant embryos. **(A)** Phenotypes of WT and mutant seeds within the same silique at 6 DAP. Scale bar = 50 μm. **(B)** Distribution of embryos at different developmental stages within the same silique from *emb2726-4/*+ plants at 12 DAP. **(C)** Phenotypes of the embryos in WT, *emb2726-4*, and *emb2726-5* seeds at different developmental stages. At the 16-cell stage, WT, and mutant embryos were indistinguishable. Scale bar = 10 μm.

To investigate the mechanism of embryonic lethality in the *EMB2726* loss-of-function mutant, seeds from *emb2726-4/*+ and *emb2726-5/*+ siliques were dissected, and the embryos in green and white seeds from the same silique were compared side by side using the whole-mount clearing technique. In siliques at 7 DAP, while the embryos in green seeds (labeled as WT-Like as shown in Figures 2A,C) were mostly at the torpedo stage, the embryos in albino seeds were arrested at the globular stage (Figure 2A). At 10 DAP, around 89% of the embryos in green seeds had reached the mature stage, and 11% were at the bent cotyledon stage (*n* > 1,000), while all of the embryos in the albino seeds (*n* > 300) were still in the globular stage without differentiation (Figure 2B). Careful observation showed that all of the embryos in *emb2726/*+ siliques developed at a similar rate and were indistinguishable from each other until the 16-cell stage, at which the embryos exhibited an outermost protodermal layer with upper- and lower-tier cells (Figure 2C). When the embryos reached the 32-cell stage, differences between the *emb2726* and WT embryos became obvious. In *emb2726* embryos, the division patterns of the two hypophyseal daughter cells were similar to those in WT embryos. Division of the protodermal cells seemed to be unaffected, but the cells were swollen. For the inner cells, although the precursors of procambium and ground tissue could be identified, the cell pattern in the mutant embryos was not as organized as in WT embryos, possibly because the cells were swollen. At 5 DAP, the embryos in green seeds could be clearly distinguished from those in the white seeds. Most of the embryos in the green seeds had reached the heart stage, but the development of the mutant embryos was arrested at the globular stage. At 12 DAP, whereas the embryos in the green seeds had reached maturity, the embryos in the white seeds remained spherical in shape. An analysis of the cellular organization pattern showed that the cells in the mutant embryos divided irregularly starting at the 32-cell stage and that the embryos grew radially without establishing bilateral symmetry (Figure 2C). Thus, the transition of the *emb2726* embryos from the globular stage to the heart stage was disrupted at the early- to mid-globular stage, confirming that EMB2726 is required for normal embryo development in *Arabidopsis*.

### EMB2726 Is a Plastid-Localized Protein

**FIGURE 3:**
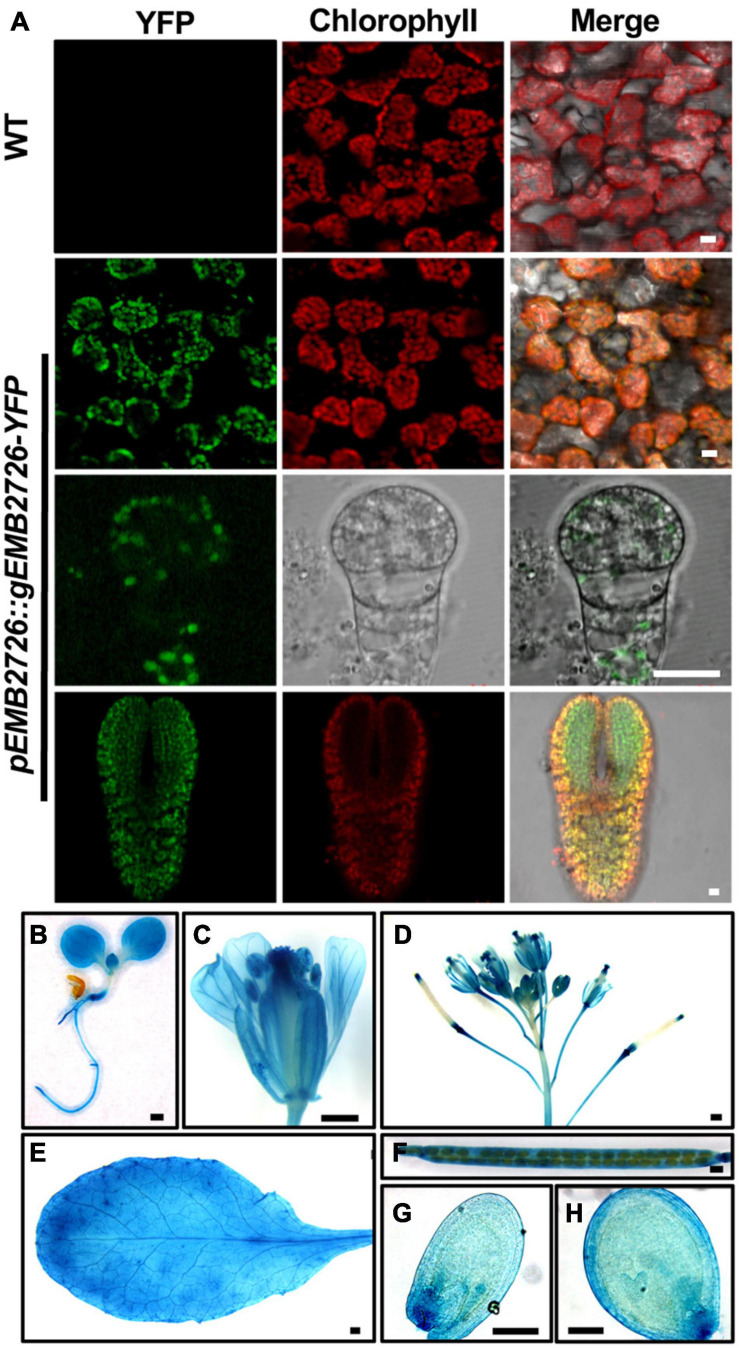
Subcellular and tissue localization of EMB2726. **(A)** Localization of EMB2726 using a *pEMB2726:gEMB2726-YFP* stable transgenic line. The top two rows show the subcellular localization of EMB2726-YFP in mesophyll cells with WT as control (top row). The third and fourth rows show the localization in octant and torpedo stage embryos, respectively. Scale bar = 10 μm. **(B–H)** GUS staining results using *pEMB2726:gEMB2726-GUS* transgenic plants. **(B)** A 7-day-old seedling; **(C)** flower; **(D)** inflorescence; **(E)** rosette leaf; **(F)** mature silique; **(G)** early globular seed; and **(H)** heart stage seed. Scale bar = 0.5 mm **(B–F)** and Scale bar = 0.1 mm **(G,H)**.

EMB2726 was suggested to be a homolog of the plastid-localized PETs protein from *C. reinhardtii*. To investigate the localization of EMB2726 in *Arabidopsis*, we observed the fluorescent signals from EMB2726-YFP in *emb2726-4/*+ leaf mesophyll cells. Confocal images showed that the YFP signals overlapped nicely with chlorophyll autofluorescence, indicating that EMB2726-YFP was localized within chloroplasts (Figure 3A, second row). Since the disruption of EMB2726 resulted in an embryo-lethal phenotype, we also analyzed the tissue and subcellular localization patterns of EMB2726-YFP in developing embryos. As shown in Figure 3A, YFP signals were observed in embryos as early as the octant stage (Figure 3A, third row), before mature chloroplasts had developed. In embryos at the torpedo stage, YFP signals were observed in cells with or without chlorophyll (Figure 3A, bottom row). Thus, EMB2726 functions not just in chloroplasts but also in multiple plastid types.

We also expressed a *pEMB2726:gEMB2726-GUS* construct in Col-0 plants, and homozygous transgenic plants were stained for GUS expression. As expected, EMB2726-GUS was expressed in all tissues examined, such as embryos and roots, implying that EMB2726 is ubiquitously expressed and functions throughout the entire seedling (Figures 3B–H).

### The EF-Ts Domains of EMB2726 Are Critical for Its Function

**FIGURE 4:**
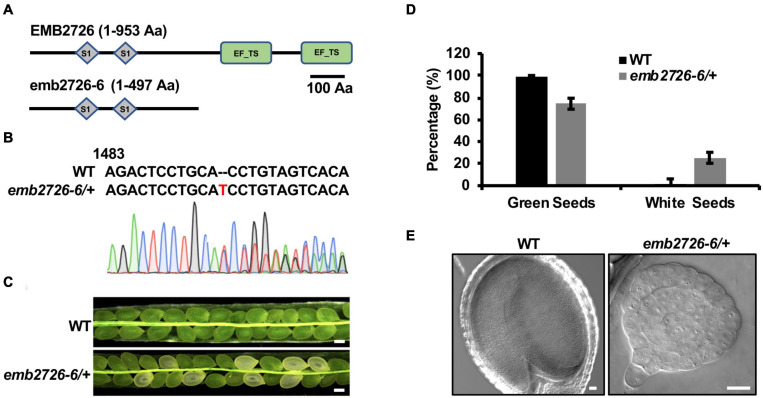
The EF-Ts domains are critical for the function of EMB2726. **(A)** Schematic diagrams of the full-length EMB2726 protein (top) and EF-Ts domain-deleted version of EMB2726 generated using the CRISPR/Cas9 system (bottom). **(B)** Sequencing chromatogram of the mutated *emb2726-6/*+ sequence generated by CRISPR/Cas9. The inserted nucleotide is highlighted in red. **(C)** Seeds in siliques from WT and *emb2726-6/*+ plants at 12 DAP. Scale bar = 0.2 mm. **(D)** Percentages of green and white seeds in WT and *emb2726-6/*+ siliques as shown in **(C)**. **(E)** The embryo phenotypes in green (left) and white (right) seeds at 12 DAP. Scale bar = 20 μm.

Polyprotein of EF-Ts protein can be post-translationally spliced into a plastid-specific ribosomal protein-7 (PSRP-7) and an EF-Ts protein (Yamaguchi et al., 2002; Belign et al., 2004). PSRP-7, which contains two S1 domains, functions as an RNA-binding protein in the ribosomal small subunit, while the EF-Ts protein functions as a GTP-GDP exchange factor for EF-Tu (Belign et al., 2004). EMB2726, a homolog of PETs, also contains two S1 domains at the amino end and two EF-Ts domains at the carboxyl end, but no post-translational splicing has been observed (Belign et al., 2004). To determine whether the EF-Ts domains play a role in embryogenesis, we generated a mutant from which these domains were deleted (named *emb2726-6*) using the CRISPR/Cas9 technique (Figures 4A,B). Similar to *emb2726-4/*+ and *emb2726-5/*+, no homozygous plants could be recovered from *emb2726-6/*+. An analysis of siliques at 10 DAP from T3 *emb2726-6/*+ seedlings revealed a similar percentage (25.3%, *n* > 800) of albino seeds, and the embryos in these seeds were all arrested at the globular stage as in the other two null mutants (Figures 4C–E). These results indicate that the EF-Ts domains of EMB2726 are critical for embryo development.

### Chloroplast Development Is Impaired in *emb2726* Embryos

**FIGURE 5:**
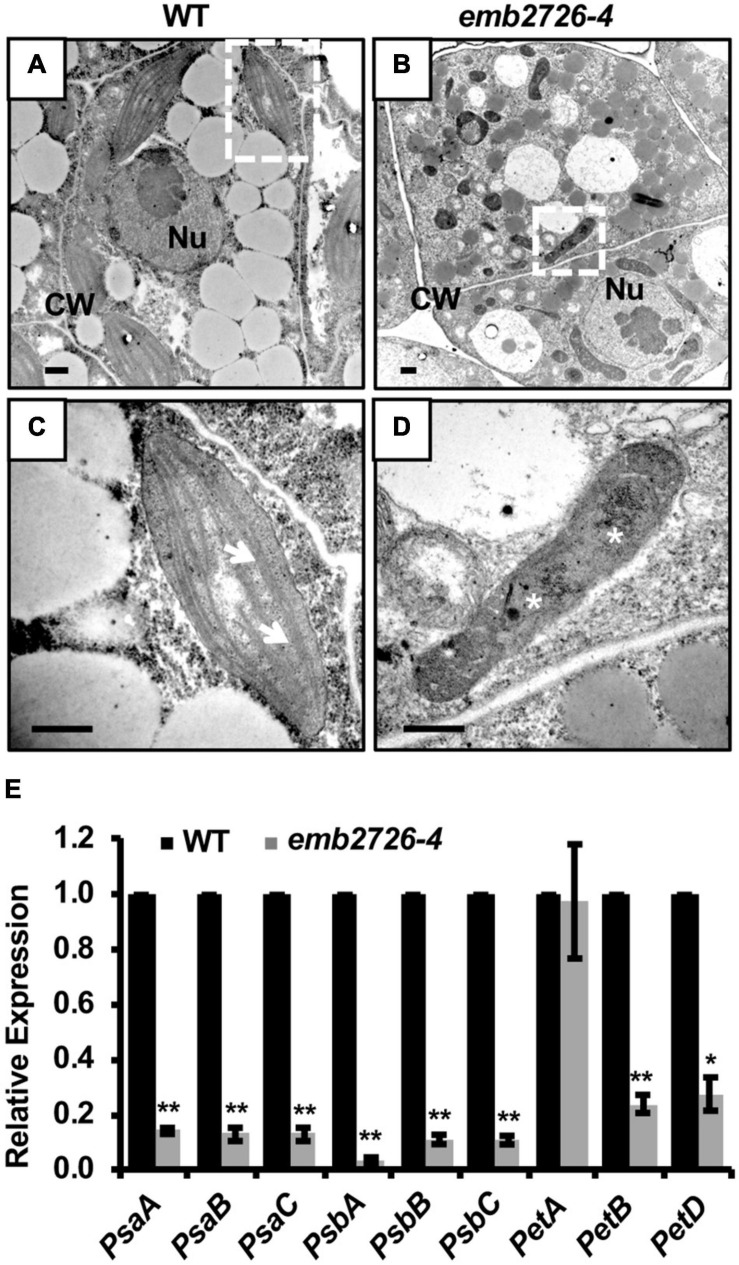
Chloroplast development is disrupted in *emb2726-4* embryo cells. **(A,B)** Cells in WT and *emb2726-4* embryos at 5 DAP. Nu, nucleus; CW, cell wall. **(C,D)** Enlarged images of the plastids in white boxes in **(A,B)**, respectively. Arrows show the grana and asterisks show the possible phytoferritin aggregations. Scale bar = 0.5 μm. **(E)** The relative transcription level of plastid-encoded photosynthesis-related genes in WT and mutant embryos from *emb2726-4/*+ siliques at 7 DAP. The asterisks indicate significant differences by Student’s *t*-test using three biological repeats. *PP2AA3* was used as an internal control. *P < 0.01, **P < 0.001.

During seed development, chloroplast biogenesis starts within the cells of mid-globular embryos, and chlorophyll biosynthesis turns the color of the seeds from white to green. Since *emb2726* seeds failed to turn green, we suspected a defect in chloroplast biogenesis. By using transmission electron microscopy, we observed the chloroplast structure in WT and mutant embryos isolated from siliques at 5 DAP. In WT embryos, the chloroplasts had an organized thylakoid membrane stacked into grana (Figures 5A,C). However, the *emb2726* embryo cells contained only ovoid or irregularly shaped, undeveloped proplastids. Darkly stained aggregations, possibly phytoferritin, could be observed in some proplastids within the mutant embryo cells, but no obvious internal membrane structure could be found (Figures 5B,D). In addition, the expression levels of *psaA*, *psaB*, *psaC*, *psbA*, *psbB*, *psbC*, *petA*, *petB*, and *petD*, which are photosynthesis-related genes encoded by the plastid genome, were analyzed by qRT-PCR. The results showed that the transcript levels of all the genes except *petA* were dramatically reduced (Figure 5E). These data suggest that the mutation of *emb2726* disrupted chloroplast development, possibly because of a failure to generate thylakoid membranes. Thus, *EMB2726* is required for chloroplast development.

### The Distribution of Auxin Is Altered in *emb2726* Embryos

One striking feature of the *emb2726* embryos was the defect in transition from radial to bilateral symmetry. Given that auxin plays an important role during this process, we examined whether a disruption in auxin distribution or auxin responses occurred in *emb2726* embryos. Three auxin-related reporter lines, *DR5:GFP*, *pDRN:DRN-GFP*, and *pPIN1:PIN1-GFP*, were crossed with *emb2726-4/*+ plants. F3 homozygous reporter lines in the *emb2726-4/*+ background were selected, and WT and mutant embryos from the same silique were examined side by side for expression of the reporter genes.

**FIGURE 6:**
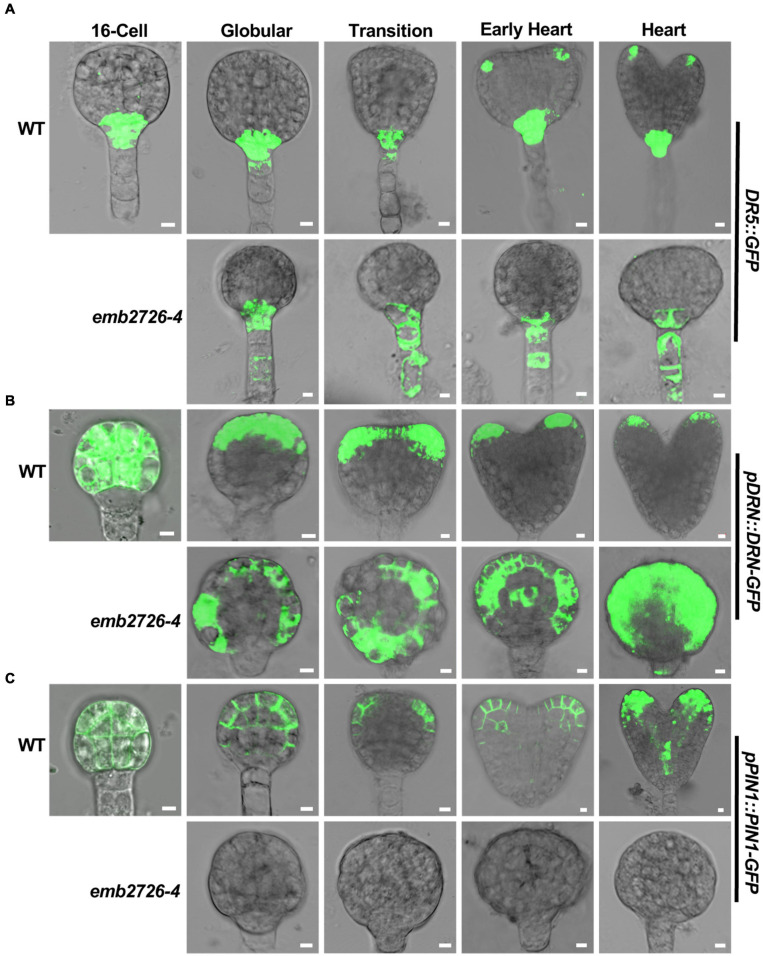
Polar auxin transport is altered in *emb2726-4* embryos. Expression patterns of *DR5:GFP* **(A)**, *pDRN:DRN-GFP* **(B)**, and *pPIN1:PIN1-GFP* **(C)** in WT and *emb2726-4* embryos at different developmental stages. A minimum of 50 embryos were observed for 16-cell stage embryos, and 30 WT and 30 mutant embryos were observed for each of the later developmental stages. Scale bar = 10 μm.

Starting from the 16-cell stage to the globular stage, auxin synthesized at the bottom of the suspensor is transported to the hypophysis and upper suspensor cells by PIN7, while auxin synthesized in the embryonic apical area is transported to the basal embryonic region by PIN1 (Möller and Weijers, 2009). Consistently, beginning from the globular stage, the DR5:GFP fluorescence in wild type was highest in hypophyseal daughter cells and sometimes in the topmost suspensor cell, but it was almost invisible in the lower suspensor cells. However, GFP signals in *emb2726* were observed in both the hypophysis and lower suspensor cells (Figure 6A). At later stages, GFP signals in WT embryos were visible at the cotyledon initiation sites and later at the tips of emerging cotyledons; however, the GFP signal in mutant embryos was restricted to the hypophysis and suspensor cells (Figure 6A). This result indicates relatively normal auxin production, and the initial differences in the distribution of DR5:GFP in the cotyledon might be due to failed differentiation of the mutant embryos at late developmental stages.

DORNROSCHEN (DRN) is involved in auxin-regulated, embryonic cotyledon development (Möller and Weijers, 2009). Consistent with a previous publication (Chandler et al., 2007, 2011), *pDRN:DRN-GFP* was expressed in all cells of both WT and *emb2726-4* embryos before the early globular stage, during which the two types of embryos were not clearly distinguishable (Figure 6B, 16-cell). Once the WT embryos reached the globular stage, DRN-GFP starts to redistribute to the apical region and then at the tip of the embryonic cotyledon; then, its expression dropped gradually, becoming almost invisible at the torpedo stage. However, within the same silique, the DRN-GFP signal in mutant embryos was maintained throughout the entire embryo as in the early globular stage (Figure 6B, *emb2726-4*). This result is consistent with the lack of cotyledon initiation in the mutant embryos.

Similar to DRN-GFP, expression of PIN1-GFP was also observed before the 16-cell stage in all embryos from *emb2726-4/*+ plants. Of more than 508- to 16-cell embryos observed from *emb2726-4/*+ siliques, only one embryo did not have any GFP fluorescence. This ratio is dramatically lower than the 25% homozygous mutants in *emb2726-4/*+, indicating that PIN1-GFP was expressed in *emb2726-4* embryos earlier than the 16-cell stage. Beginning at the 32-cell stage, no PIN1-GFP fluorescence could be observed in the mutant embryos (Figure 6C, *emb2726-4*), while WT embryos from the same siliques displayed the correct distribution pattern, as reported previously (Lau et al., 2012). Together with the findings for *DR5:GFP* and DRN-GFP, the results indicate that the auxin redistribution, instead of a change in auxin biosynthesis or metabolism, contributes to the defective development of *emb2726* embryos.

### Initiation of the Procambium and Ground Tissue Is Disrupted in the Mutant Embryos

**FIGURE 7:**
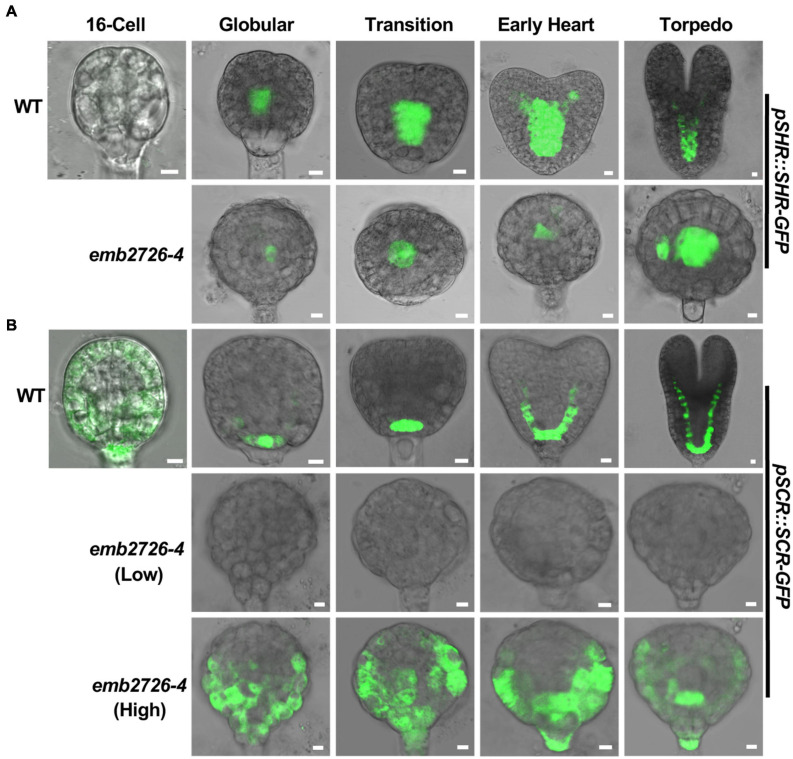
Initiation of the procambium and the ground tissue is disrupted in *emb2726-4* embryos. Expression patterns of *pSHR:SHR-GFP* **(A)** and *pSCR:SCR-GFP* **(B)** in WT and *emb2726-4* embryos at different developmental stages. The terms “Low” and “High” in parentheses indicate the excitation power used. A minimum of 50 embryos were observed for 16-cell stage embryos, and 30 WT and 30 mutant embryos were observed for each of the later developmental stages. Scale bar = 10 μm.

In addition to auxin-related reporters, we examined whether the procambium and the ground tissue were present in *emb2726* embryos using F3 homozygous *pSHR:SHR-GFP* and *pSCR:SCR-GFP* reporter lines in an *emb2726-4/*+ background. Consistent with previous publications, in WT embryos, SHR-GFP fluorescence was observed in procambium precursor cells at the early globular stage and was later expanded to the newly formed stele at the early heart stage (Figure 7A, WT). Meanwhile, SCR-GFP fluorescence was observed in hypophyseal cells and then in the newly formed ground tissue as the embryos continued to develop (Wysocka-Diller et al., 2000) (Figure 7B, WT). In *emb2726-4* embryos, SHR-GFP expression was observed at the globular stage, indicating that procambium precursor cells were present. When the WT embryos reached the torpedo stage, although the mutant embryos grew bigger, the SHR-GFP expression was limited to the same position (Figure 7A, *emb2726-4*). In comparison, SCR-GFP fluorescence was not observed in *emb2726-4* embryos starting from the early globular stage when using the same excitation power as for WT embryos (Figure 7B). However, if much higher excitation power was used, very weak, diffused SCR-GFP fluorescence was observed. Unlike the WT embryos, in which SCR-GFP was restricted to the hypophysis, the signal was spread through the lower portion of *emb2726* embryos, indicating that SCR-GFP was misexpressed at a low level. These results suggest that, although *emb2726-4* embryos could continue to grow, the procambium and the ground tissue are not formed, possibly because the expression of SCR-GFP is too weak and mislocalized.

## Discussion

### EMB2726 May Function as an EF-Ts to Regulate Plastid Translation

In prokaryotes, EF-Ts functions as a guanine nucleotide exchange factor for EF-Tu during protein synthesis. Without EF-Ts, EF-Tu remains in the inactive EF-Tu⋅GDP form, and translation is inhibited. On the website of *Arabidopsis* Information Resource Center^1^, three genes were annotated as a possible EF-Ts. One of them (*At4g11120*) is localized in mitochondria, and two (*At2g25800* and *EMB2726*) are localized in chloroplasts. However, an analysis of the At2g25800 protein sequence revealed neither an EF-Ts motif nor homology with known EF-Ts, indicating misannotation of the protein and leaving EMB2726 as the only possible EF-Ts in chloroplasts. It has been shown that the homolog of EMB2726 in *C. reinhardtii*, PETs protein, can be post-translationally processed into PSRP-7 and an EF-Ts protein and that the latter functions as a guanine nucleotide exchange factor for EF-Tu (Belign et al., 2004). However, EMB2726 in *Arabidopsis* seems to lack this post-translational process, and more than 80% of the proteins translated contain two EF-Ts domains (Belign et al., 2004). The immunoblotting result from *pEMB2726:EMB2726-GFP* complementation lines confirmed that the full-length protein containing two EF-Ts domains was the major form synthesized (Figure 1F). In addition, the *emb2726-6/*+ mutant created by deleting the two EF-Ts domains showed phenotypes similar to those of two null mutants, *emb2726-4/*+, and *emb2726-5/*+. These results suggest that the EF-Ts domains are crucial for the function of EMB2726 in *Arabidopsis*. Since *EMB2726* encodes the only possible EF-Ts that functions in *Arabidopsis* plastids, it is safe to assume that EMB2726 functions as a guanine nucleotide exchange factor during plastid translation.

Plastid protein translation is required for embryogenesis in *Arabidopsis* (Meinke, 2020). Disruption of protein biosynthesis in plastids, such as the knockout of important proteins that function in plastid ribosomes (Yin et al., 2012) or plastid translation processes (Ruppel and Hangarter, 2007), produces similar plastid and embryo phenotypes as in *emb2726*. In *Arabidopsis*, except for photosynthesis-related genes, most plastid-encoded genes function in transcription or translation. However, the protein (or proteins) whose misexpression is responsible for the observed embryonic lethal phenotype is unclear. The plastid-encoded protein accD, a subunit of the heteromeric enzyme acetyl-CoA carboxylase in plastids and which is required for membrane lipid biosynthesis, is a leading candidate (Bryant et al., 2011; Meinke, 2020). Interestingly, mutants of the other two subunits of this enzyme, as well as the biological processes that require functional accD, all displayed embryonic lethality (Li et al., 2011). Further, *emb2726* and other plastid translation mutants have a common phenotype: a lack of internal membrane structure in plastids. In addition, a suppressor/enhancer screen for mutants with embryos arrested at the mid-globular stage identified *ACC2*, a nucleus-encoded plastid-localized homomeric acetyl-CoA carboxylase gene, possibly because ACC2 could produce low-level fatty acids in plastids (Parker et al., 2014; Meinke, 2020). Although these findings suggest that accD is critical for embryogenesis, direct genetic evidence from accD knockout mutants is required to prove that the lack of accD results in embryonic lethality.

### EMB2726-Supported Plastid Development Is Required for Embryogenesis

During embryo development in *Arabidopsis*, the globular stage is a critical developmental stage that marks the transition of the embryo from cell division to organ initiation. During this transition, an important event is chloroplast development. Mutants impaired in plastid biogenesis but not chlorophyll biosynthesis often show arrested embryo development (Bryant et al., 2011; Liu et al., 2017; Meinke, 2020). In this study, we found that *EMB2726* is required for plastid and embryo development. The lack of green coloration and the reduced expression of photosynthesis-related genes in *emb2726-4* seeds indicate a lack of chloroplast development. Transmission electron microscopy showed that, unlike the plastids from WT embryos that developed under conditions of light deprivation, which exhibited an obvious prolamellar body and pre-thylakoid membranes (Liu et al., 2017), the plastids in embryonic *emb2726-4* cells remained at the proplastid stage. These results confirm that the lack of green coloration was not simply due to the lack of photosynthesis compartments, but rather failure in plastid biogenesis, and they demonstrate that functional EMB2726 is required for chloroplast development.

A common phenotype of *emb2726* and strong plastid biogenesis mutants is that the embryo cells are able to divide continuously, resulting in a spherical embryo with a mass of cells (Yin et al., 2012; Ye et al., 2017; Chen et al., 2018) or an abnormal heart-shaped embryo (Chen et al., 2015). The developmental defect in *emb2726* embryos could be observed as early as the 32-cell stage, and the defective embryo cells were able to divide for several rounds. This cell division ability indicates that plastid differentiation is not essential for cell viability and division. However, after the 32-cell stage, cell division in *emb2726* embryos occurred incorrectly, leading to an abnormal cellular pattern without organ initiation. Thus, EMB2726-mediated plastid development is crucial for embryonic organogenesis. This was supported by the observation that proplastids remain undifferentiated from the zygote to the globular stage (Mansfield and Briarty, 1991). However, the first three to four rounds of cell division occurred with the correct pattern in *emb2726* embryos. Whether this was because proplastids are not required for these divisions or that proplastids possess a minimum ability to support these divisions requires further investigation.

It remains unclear how plastids control the transition from the globular to the heart stage. As photosynthesis is not required for this transition, other plastid functions, such as synthesis of fatty acids, retrograde signaling molecules, and hormone precursors, may play crucial roles. As discussed earlier, a lack of accD could cause a deficiency in lipid biosynthesis. Lipids are not only the building blocks of membranes, they can also function as signaling molecules to maintain membrane electrostatics or can be required for signaling processes or enzyme activity (Boutté and Jaillais, 2020). The functional diversity of membrane lipids could affect multiple cellular growth and developmental processes that govern embryogenesis. Another possibility is retrograde signaling. Retrograde signals are important to integrate environmental stresses during plant development (de Souza et al., 2017). Whether they also play a role in embryo development is currently unknown. Support for this idea comes from studies of the heme biosynthesis enzyme ferrochelatase 1 (FC1). Heme is one of the retrograde signals generated by plastids, and FC1, which catalyzes the last step of heme biosynthesis, is embryo-lethal when mutated (Chan et al., 2016; Fan et al., 2019). However, instead of being arrested at the globular stage, *fc1* embryos do not show any abnormalities until the torpedo stage (Fan et al., 2019). Currently, it is unclear whether plastid-originated molecules regulate embryo development independently or collectively, or whether plastids regulate various developmental stages *via* different mechanisms. Genetic and complementation tests are required to answer these questions.

### EMB2726 Affects the Auxin Distribution and Pattern Formation in Developing Embryos

During *Arabidopsis* embryogenesis, protoderm, ground tissue, and procambium initiation, and root meristem precursor cell determination happen at the globular stage (Lau et al., 2012). It is well known that auxin plays an indispensable role during *Arabidopsis* embryo pattern formation (Locascio et al., 2014; Smit and Weijers, 2015; Verma et al., 2021). In *emb2726-4* embryos, PIN1 expression was not observed, and DR5:GFP fluorescence was seen in both the hypophysis and lower suspensor cells, similar to the *pin7* mutant (Robert et al., 2013), indicating that polar auxin transport was disrupted in *emb2726* embryos. Moreover, instead of being restricted to the apical region at the globular and transition stages, the expression of DRN, whose transcription is activated by the auxin response factor MONOPTEROS and whose protein affects auxin transport (Cole et al., 2009; Verma et al., 2021), was detected throughout *emb2726* embryos, suggesting that the distribution of auxin was disturbed in the mutant.

During embryonic root development, initiation of the root apical meristem starts with the asymmetric division of the hypophysis. In *emb2726* embryos, the initiation of root patterning was unaffected because of the evidently visible lens-shaped hypophyseal daughter cell. A possible explanation for this is that the auxin response, as indicated by DR5-GFP fluorescence, remained strongest in the hypophysis and its daughter cells. Despite the overall disrupted auxin distribution, this localized auxin concentration might still be sufficient for hypophysis specification. However, the arrested root meristem development in *emb2726* embryos indicates that this localized auxin concentration is not high enough or that additional factors are required. In addition to auxin, the SHR-SCR transcription network is important for root apical meristem development. SCR is required for the division of quiescent center (QC) and columella precursors but not for initial hypophyseal division (Wysocka-Diller et al., 2000). In *emb2726* embryos, the lens-shaped QC precursor cell did not differentiate further and could not be maintained at later stages; thus, the root cap did not form. This phenotype is consistent with the low-level expression of SCR-GFP in *emb2726* embryos. Therefore, another possible reason for the failure of root initiation in *emb2726* could be the misexpression of SCR in hypophyseal daughter cells.

Nonetheless, the SHR-SCR transcription network is not required for ground tissue initiation; instead, auxin could guide the specification of the first ground tissue cells through MONOPTEROS/ARF5 in an SHR-independent manner (Möller et al., 2017). Furthermore, the initiation of vascular tissue is mediated by auxin (Ohashi-Ito et al., 2013). Thus, the disrupted division of the ground tissue and vascular initial cells in *emb2726* embryos might be exclusively due to the disturbed distribution of auxin.

Overall, in *emb2726* embryos, a disrupted auxin distribution was observed after the 16-cell stage, and disrupted cell patterning was distinctly visible at the 32-cell stage. These findings support the notion that the arrested embryos observed in *emb2726* were mainly caused by the disrupted distribution of auxin. Still, factors unrelated to auxin might also be involved in the patterning of *emb2726* embryos. For instance, the involvement of auxin in protodermal cell fate has not been reported, but the periclinal division was observed in the protoderm layer as *emb2726* embryos grew larger (Supplementary Figure 1). However, how EMB2726 affects the function of such factors and the expression of PINs is unclear.

## Conclusion

The results show that EMB2726 is crucial for the globular to the heart stage transition during *Arabidopsis* embryo development. Disruption of the function of EMB2726 could lead to a loss of EF-Ts activity during plastid translation, leading to failed embryogenesis.

## Data Availability Statement

The original contributions presented in the study are included in the article/Supplementary Material, further inquiries can be directed to the corresponding author.

## Author Contributions

CL performed most of the experiments and analyzed the data. J-XS and CQ participated in the phenotype analysis. BZ, JW, and SW participated in the analysis of the reporter lines. YS designed the experiments and wrote the manuscript. All authors made substantial, direct and intellectual contributions in the study, and approved it for publication.

## Conflict of Interest

The authors declare that the research was conducted in the absence of any commercial or financial relationships that could be construed as a potential conflict of interest.

## Publisher’s Note

All claims expressed in this article are solely those of the authors and do not necessarily represent those of their affiliated organizations, or those of the publisher, the editors and the reviewers. Any product that may be evaluated in this article, or claim that may be made by its manufacturer, is not guaranteed or endorsed by the publisher.

## Footnotes

1 www.arabidopsis.org
