# Supplementary material for: Plastid-Localized EMB2726 Is Involved in Chloroplast Biogenesis and Early Embryo Development in Arabidopsis
Source: Front Plant Sci. 2021 Jul 23;12:675838. doi: 10.3389/fpls.2021.675838 (PMC8343077; doi:10.3389/fpls.2021.675838)
Supplement: Supplementary file 1 [file Table_1.pdf]

Supplementary Table 1: Primers used in this study

Gene number	Primer sequence (5'-3')	Purpose
At4g29060 (*EMB2726*)	TATAGGGCAAGCCTTTGGTG	*emb2726-4* identification
	CAAGTGTTTCCGTTTGCTCTTC	
	CACCATGGCTACGATTACACCTTC	*emb2726-5* identification
	CAAGTGTTTCCGTTTGCTCTTC	
	GTGAAGAGGAAATTGTTGAGAAC	*emb2726-6* identification
	TTCTTACCTGTGGATTAGCC	
	TCTCTGCCACAAACTCTCTCATTC	*proEMB2726::EMB2726*, entry
	GTTATCTTCTCCAAGAGTAAACTTCACAA	
	ATATATGGTCTCGATTGGAAGCTCCAGGAATGAGCCGTT	*emb2726-5*, Crispr
	TGGAAGCTCCAGGAATGAGCCGTTTTAGAGCTAGAAATAGC	
	AACCAGGCATCTCACTCTTCCTCAATCTCTTAGTCGACTCTAC	
	ATTATTGGTCTCGAAACCAGGCATCTCACTCTTCCTC	
	ATATATGGTCTCGATTGCTTCTGTGACTACAGGTGCGTT	*emb2726-6*, Crispr
	TGCTTCTGTGACTACAGGTGCGTTTTAGAGCTAGAAATAGC	
	AACGCCCCTGTTTCTTCTCTCACAATCTCTTAGTCGACTCTAC	
	ATTATTGGTCTCGAAACGCCCCTGTTTCTTCTCTCAC	
ATCG00350 (*PsaA*)	GGGCAGGACATCAAGTACAT	qRT-PCR
	AGCCAAAAGATCCCGATTCA	
ATCG00340 (*PsaB*)	GCCAAGGCTTAGCTCAGGAC	qRT-PCR
	CCCGAAATGAGAAGCAAAAA	
ATCG01060 (*PsaC*)	ATAGGATGTACTCAATGTGT	qRT-PCR
	TCTCTTACAACCAACACAGT	
ATCG00020 (*PsbA*)	GCGAAAGCGAAAGCCTATGG	qRT-PCR
	CAATGAATGCGATAATAAAA	
ATCG00680 (*PsbB*)	GGCGTGGAGGGTTTTGAC	qRT-PCR
	GGCGGACGAACACTAAGATG	
ATCG00280 (*PsbC*)	ACGCTCTTTAATGGAACTTT	qRT-PCR
	TTAATCCGGCATGGGCTACA	
ATCG00540 (*PetA*)	CCCGAGATGAAAGAAAAGAT	qRT-PCR
	GGAGCAAGAATAGGAAAGGT	
ATCG00720 (*PetB*)	GATGGTCGGCAAGTATGATGG	qRT-PCR
	GGTCAATACACCCAGAACCACA	
ATCG00730 (*PetD*)	TTTCAGTACCAGCGGGATTAT	qRT-PCR
	GTTGCTCCAATACCTAACCACA	
At1G13320 (*PP2AA3*)	TTCTCGCTCCAGTAATGGGATCCGA	qRT-PCR
	GTTCTCCACA ACCGCTTGGT CGACT	
