# Supplementary material for: Plastid-Localized EMB2726 Is Involved in Chloroplast Biogenesis and Early Embryo Development in Arabidopsis
Source: Front Plant Sci. 2021 Jul 23;12:675838. doi: 10.3389/fpls.2021.675838 (PMC8343077; doi:10.3389/fpls.2021.675838)
Supplement: Supplementary file 2 [file Table_2.pdf]

Supplementary Table 2: Reciprocal cross results between *emb2726-4/+* and WT

Parental genotype	Progeny genotype						
**Female x Male**	**WT**	**HETE**	**HOMO**	**TE_M**	**TE_F**	**X^2**	**P-value**
***emb2726-4/+* self**	137	231	0	—	—	126.52	<0.0001
**WT x *emb2726-4/+***	77	66	NA	85.71%	NA	0.846	0.357
***emb2726-4/+* x WT**	115	107	NA	NA	93.04%	0.288	0.591

Reciprocal crosses were performed between *emb2726-4/+* and WT plants. The transmission efficiency (TE) of gametes was calculated as follows: TE=number of heterozygote progenies/number of wild type progenies X 100%. P-values were calculated using the x^2 test. TE_F, female transmission efficiency; TE_M, male transmission efficiency; NA, not applicable.
