# Supplementary material for: Plastid-Localized EMB2726 Is Involved in Chloroplast Biogenesis and Early Embryo Development in Arabidopsis
Source: Front Plant Sci. 2021 Jul 23;12:675838. doi: 10.3389/fpls.2021.675838 (PMC8343077; doi:10.3389/fpls.2021.675838)
Supplement: Supplementary file 3 [file Data_Sheet_1.PDF]

Figure S1

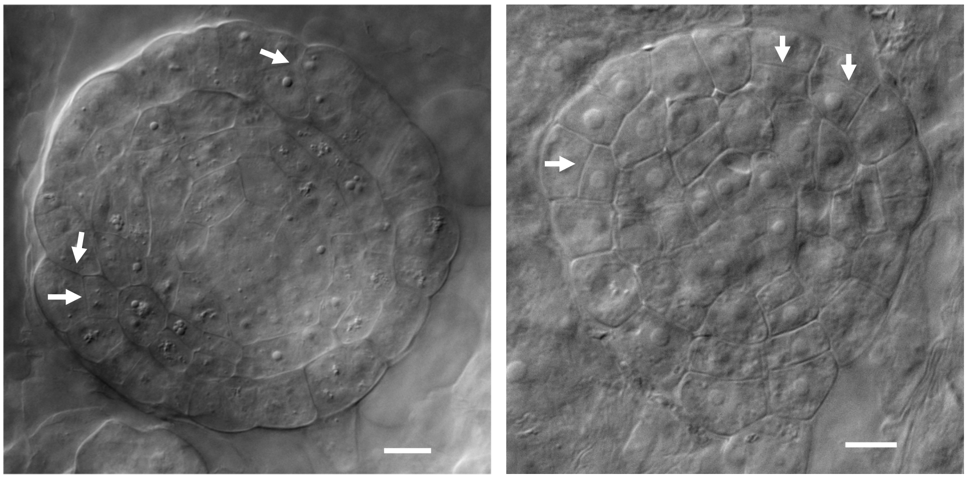

Supplementary Figure 1. Representative terminal embryo phenotype of *emb2726*. Arrows pointing to abnormal division plane in protodermal layer. Scale bar=20 μm
